# IL-10 to lymphocyte ratio (ILR) and lactate in the prognosis prediction and risk stratification of sepsis: a pilot study

**DOI:** 10.3389/fmed.2025.1665915

**Published:** 2025-09-10

**Authors:** Yuan Yan, Xiao Liu, Xiaoying Fu, Jing Qin, Faming He, Bin Liu, Bailin Niu

**Affiliations:** ^1^Department of Intensive Care Medicine, Chongqing Emergency Medical Center, Chongqing University Central Hospital, School of Medicine, Chongqing University, Chongqing, China; ^2^Department of Emergency and Critical Care Medicine, The First Affiliated Hospital of Chongqing Medical University, Chongqing, China

**Keywords:** sepsis, interleukin-10, lymphocyte count, lactate, prognosis prediction, risk stratification

## Abstract

**Background:**

Sepsis is a highly heterogeneous clinical syndrome, and the real-time prognosis prediction and risk stratification for it remain a big challenge in current clinical research. This study aimed to assess the performance of IL-10/lymphocyte ratio (ILR) and lactate (Lac) in the prognostic prediction and risk stratification of sepsis.

**Methods:**

This is a retrospective observational study that included 148 patients with sepsis admitted to the First Affiliated Hospital of Chongqing Medical University from January 2022 to February 2023. Data collection commenced on the first day of ICU admission, with clinical and laboratory parameters recorded within 24 h of diagnosis, including IL-10 levels, lymphocyte counts, Lac, SOFA score, and APACHE II score. The relationship between ILR and Lac and 28-day mortality were analyzed by multivariate logistic regression analysis and Cox proportional hazards regression, and their predictive efficacy were assessed by receiver operator characteristic curves (ROCs), and Kaplan–Meier survival curves were used to validate the effect of risk stratification.

**Results:**

Patients in the death group exhibited significantly higher ILR (302.33 vs. 16.37) and Lac levels (3.25 mmol/L vs. 1.90 mmol/L) compared to the survival group (both *p* < 0.001). Multivariate logistic regression analysis showed that ILR (OR = 1.005, 95% CI 1.001–1.009) was independent risk factor for death at 28 days. Analysis of ROCs showed that the predictive efficacy of ILR (AUC = 0.860) was superior to the APACHE II score (AUC = 0.797) and the SOFA score (AUC = 0.704). Based on stratification by ILR (cutoff value 97.4) and Lac (cutoff value 4.1 mmol/L), the four risk stratification levels (Levels I–IV) exhibited progressively decreasing 28-day mortality rates: Level I (78.95%), Level II (50.00%), Level III (15.38%), and Level IV (7.69%). Kaplan–Meier analysis confirmed significant survival differences (*p* < 0.001), with Level I demonstrating the worst prognosis.

**Conclusion:**

The combined ILR and Lac measurement provides a practical bedside tool for real-time sepsis risk stratification, demonstrating better prognostic utility than conventional scoring systems while maintaining clinical feasibility.

## Highlights

- Uniquely combines Interleukin-10-to-Lymphocyte Ratio (ILR) and lactate for sepsis prognosis (AUC = 0.860), outperforming SOFA and APACHE II scores.
- Risk stratification using optimal cutoffs (ILR ≥ 97.4; Lactate ≥4.1 mmol/L) identified four distinct risk groups (I–IV) with statistically significant gradients in mortality rates at both 7-day (57.9 to 3.9%) and 28-day follow-ups (78.9 to 7.7%) (*p* < 0.001).
- Leverages readily available, low-cost routine tests with minimal iatrogenic burden, enabling real-time, repeatable risk assessment at the bedside—unlike complex multi-parameter or big-data models.

## 1 Introduction

Sepsis is a life-threatening organ dysfunction caused by a dysregulated host response to infection (1), and as one of the leading causes of death in critically ill patients worldwide, the complexity and clinical heterogeneity of its pathophysiologic mechanisms pose a great challenge for accurate prognostic assessment (2, 3). Sepsis manifests itself as a “cytokine storm” with over-activation of pro-inflammatory responses in the early stages. In the later stages, it turns into a predominantly immunosuppressive pathology. This immune imbalance persists throughout the disease, which is a key factor leading to organ failure and death (4, 5). Although predictive models based on artificial intelligence and multi-omics technologies have shown potential in sepsis typing and risk stratification in recent years, these methods generally suffer from complexity, high cost, and poor clinical accessibility, making it difficult to generalize their use in routine medical practice. Meanwhile, conventional scoring systems (e.g., SOFA and APACHE II), although widely used, have insufficiently reflected the immunosuppressive state, a key pathological feature of sepsis, limiting their application in accurate prognostic prediction (6).

The anti-inflammatory cytokines interleukin-10 (IL-10) and lymphocyte depletion are two key features in the immunosuppressive mechanisms of sepsis (7, 8). IL-10 is one of the most important anti-inflammatory cytokines and is secreted by monocytes/macrophages and T-cell subsets, which are capable of inhibiting the release of pro-inflammatory factors (e.g., TNF-α, IL-6) and suppressing effector T-cell function (9, 10). Studies have shown that IL-10 levels are significantly elevated in septic patients early in life and persist for a long period, and its concentration is positively correlated with mortality (11, 12). On the other hand, lymphopenia is another central manifestation of sepsis immunosuppression involving apoptosis or dysfunction of T and B cells (13, 14), and persistently low lymphocyte counts (<0.76 × 10^9^/L) for more than 3 days were significantly associated with 28-day mortality (15, 16). In addition, elevated levels of lactate (Lac), a product of hypoxic metabolism, directly reflect tissue hypoperfusion and mitochondrial dysfunction, and are closely associated with septic multi-organ failure (17).

Based on the above background, the present study innovatively proposes to combine IL-10/lymphocyte ratio (ILR) and Lac as a novel composite biomarker. This strategy has the following scientific rationale and clinical significance: the ILR reflects both the intensity of the anti-inflammatory response (via IL-10 levels) and quantifies the degree of immune cell depletion (via lymphocyte counts), thus providing a more comprehensive assessment of the immunosuppressive state of sepsis. While previous studies demonstrated that both elevated IL-10 and lymphopenia are independently associated with sepsis mortality (18, 19), their combination appears to provide enhanced predictive value. Lac serves as a marker of tissue hypoperfusion, and elevated Lac levels directly reflect the severity of tissue hypoxia and organ dysfunction. The synergistic effect of Lac with immunosuppressive indicators (e.g., IL-10) may further exacerbate organ failure, so the inclusion of Lac in and indicators may reflect the pathophysiological status of septic patients more comprehensively and improve the accuracy of prognostic assessment. Finally, these three indicators are routine clinical tests with the advantages of being simple to obtain and interpret, low cost, and low iatrogenic consumption of blood during repeated real-time detections, they are particularly suitable for bedside clinical practice.

This study aims to investigate the clinical value of ILR and Lac in the prognostic assessment and risk stratification of septic patients, and to verify its feasibility as a prognostic indicator and risk stratification by analyzing its relationship with 28-day mortality rate and traditional scoring systems (e.g., SOFA, APACHE II). The significance of this study is to provide a new theoretical basis and clinical tools for the prognostic management of sepsis, to help early identification of high-risk patients, to optimize intervention strategies, and thus to improve the survival outcomes of patients.

## 2 Materials and methods

### 2.1 Study population

A total of 197 patients with sepsis who attended the First Affiliated Hospital of Chongqing Medical University between January 2022 and February 2023 were included in this study. The study was a retrospective observational study and was approved by the Ethics Committee of the First Affiliated Hospital of Chongqing Medical University (NO. 2022-K456).

### 2.2 Inclusion and exclusion criteria

Inclusion:

1. All hospitalized patients with sepsis. Sepsis was defined as the presence of a Sequential Organ Failure Assessment (SOFA) score ≥2 based on an infection (definite or suspected), i.e., indicating the presence of organ dysfunction caused by the infection.
2. Adult patients (age ≥18 years).

Exclusion:

(1) patients with a history of comorbid hematologic disease; (2) patients with a history of extensive hormone therapy; (3) patients with an unclear baseline SOFA score; (4) patients for whom complete information was not available; (5) patients for whom laboratory tests were not completed within 24 h of the patient’s admission to the hospital with a confirmed diagnosis of sepsis; and (6) patients with a history of malignant neoplasm treated with radiotherapy or chemotherapy in the 6 months before admission to the hospital.

Definition of sepsis: the presence of a Sequential Organ Failure Assessment (SOFA) score ≥2 based on an infection (definite or suspected), which is indicative of the presence of organ dysfunction caused by infection (1).

The definition of infection includes the following aspects (20, 21): (1) the presence of a systemic reaction in the body, such as fever or a drop in body temperature, accompanied by corresponding clinical symptoms, which may involve the gastrointestinal, respiratory, and urinary tracts; (2) the presence of some common indicators of infection, such as the white blood cell count, the percentage of neutrophils, the concentration of C-reactive protein, and serum calcitoninogen levels; and (3) the presence of an area or pathologic basis for suspected infection.

#### 2.2.1 Plasma IL-10 measurement

In this study, IL-10 was measured using a commercial multiplex bead-based assay kit. First, plasma samples were collected from the peripheral blood of each septic patient at the time of patient enrollment. The plasma sample collection process consisted of placing whole blood in a tube containing sodium citrate and centrifuging it in a centrifuge at 1,000 × g for 10 min to separate the plasma. After centrifugation, the upper plasma layer was removed and immediately frozen to −80 °C, in order to ensure the stability of the samples and the accuracy of the subsequent analysis. For IL-10 assay, follow the kit manufacturer’s instructions. Specific steps included thawing the frozen plasma samples and diluting them to the appropriate concentration to comply with the kit requirements. Subsequently, the diluted samples are added to a pre-prepared multiplexed bead-based assay plate, ensuring that each sample is evenly distributed. Through the use of specific fluorescent-labeled antibodies, combined with flow cytometry techniques, the concentration of IL-10 in the samples was able to be accurately determined. All samples were assayed under consistent experimental conditions to minimize experimental errors and ensure comparable results.

#### 2.2.2 Lymphocyte count measurement

In this study, lymphocyte counts were performed using EDTA anticoagulated tubes (purple tubes) to collect peripheral venous blood to avoid coagulation or hemolysis interfering with the count results. The samples were stored at 2–8 °C, and the assay was completed within 4 h to prevent changes in cell morphology from affecting the accuracy.

#### 2.2.3 Blood lactate measurement

In the present study, Lac was determined using blood gas analyzer. Blood samples were collected from the arterial blood of each septic patient at the time of patient enrollment. The samples were placed in test tubes containing anticoagulant immediately after collection to prevent blood clotting. Subsequently, the samples were stored in an ice bath and assayed within 30 min of collection to ensure the accuracy of the results. During the measurement, the operating procedures of the blood gas analyzer were strictly followed to minimize experimental errors and to ensure comparable results.

IL-10, lymphocyte count, and Lac assays were performed under the unified quality control of the Clinical Laboratory Center of the First Affiliated Hospital of Chongqing Medical University. The Interleukin-10 to lymphocyte ratio (ILR) in this study was calculated using the IL-10 concentration and absolute lymphocyte count measured within 24 h of admission. The formula used was: ILR = IL-10 (pg/mL)/lymphocyte count (×10^9^/L). Consequently, the unit of ILR is pg/10^9^.

### 2.3 Data collection

The starting time for data collection was the first day of the patient’s admission to the ICU, and all relevant laboratory data and clinical information were recorded at this time. During the data collection process, emphasis was placed on the basic demographic characteristics of the patients, including age and gender. In addition, detailed information on the patient’s medical history, such as hypertension, coronary artery disease, chronic kidney disease, diabetes mellitus, chronic obstructive pulmonary disease, chronic liver disease, and other comorbidities, was recorded. The definitions of these variables were based on internationally recognized medical standards to ensure consistency and comparability of data. Laboratory data were collected, including lymphocyte count, Lac, and IL-10 measurements. In addition, data on other relevant biomarkers such as creatinine, platelet count, Glasgow Coma Score, temperature, PCT, interleukin 6, D-Dimer, albumin, acute kidney injury, and SOFA score were also collected in order to assess the patient’s clinical status more comprehensively.

### 2.4 Statistical analysis

In this study, the collected data were statistically analyzed using SPSS 29.0, GraphPad Prism 10, and R language 4.4.0. For count data, expressed as frequencies and percentages, the chi-square test was used for comparison between groups. For the measurement data, if normal distribution was satisfied, they were analyzed by the paired *t*-test to assess the differences between the groups, and the results were reported as means and their standard deviations (*x̄*±*s*); if normal distribution was not satisfied, comparisons between the groups were made using the Wilcoxon-Mann–Whitney U-test, and the results were expressed as the median (interquartile spacing) [M(IQR)]. In addition, multivariate logistic regression analysis was utilized in this study to identify independent risk factors for death in patients with sepsis and to explore the important role of ILR in the prognostic assessment of patients with sepsis by the Cox proportional hazards regression. In order to assess the diagnostic validity of the relevant indices, a receiver operator characteristic curve (ROC) was used, and the area under the curve (AUC) was calculated to assess the predictive efficacy. Meanwhile, SHAP (Shapley Additive Explanations) value analysis was introduced by training the XGBoost model, demonstrating the key indicators affecting the prognosis of death in septic patients. Patients were categorized into four risk stratification levels (I-IV) based on the optimal cutoff value of ILR and Lac. Kaplan–Meier survival curves were used to plot the survival of different levels, and the differences between risk stratification levels were statistically compared by the Log-Rank test to further validate the application value of ILR in clinical prognostic assessment. To ensure the quality of the data, missing values were strictly handled in the data organization process of this study. First, all clinical variables included in the analysis were screened for completeness, and variables with a high percentage of missing values or limited clinical significance were not included in the modeling analysis. For the very small proportion of missing values, simple imputation was applied (median for continuous variables and mode for categorical variables). Importantly, the core predictors (ILR and lactate) and outcomes had no missing data, ensuring the reliability of the main results. Data preprocessing was mainly done in SPSS 29.0, and the R language was used to further verify the consistency of the processed data and the robustness of the analysis. All statistical results were tested for significance with a *p*-value of less than 0.05 to ensure the scientific validity and effectiveness of the study results.

## 3 Results

### 3.1 Enrolled cases

**Figure 1:**
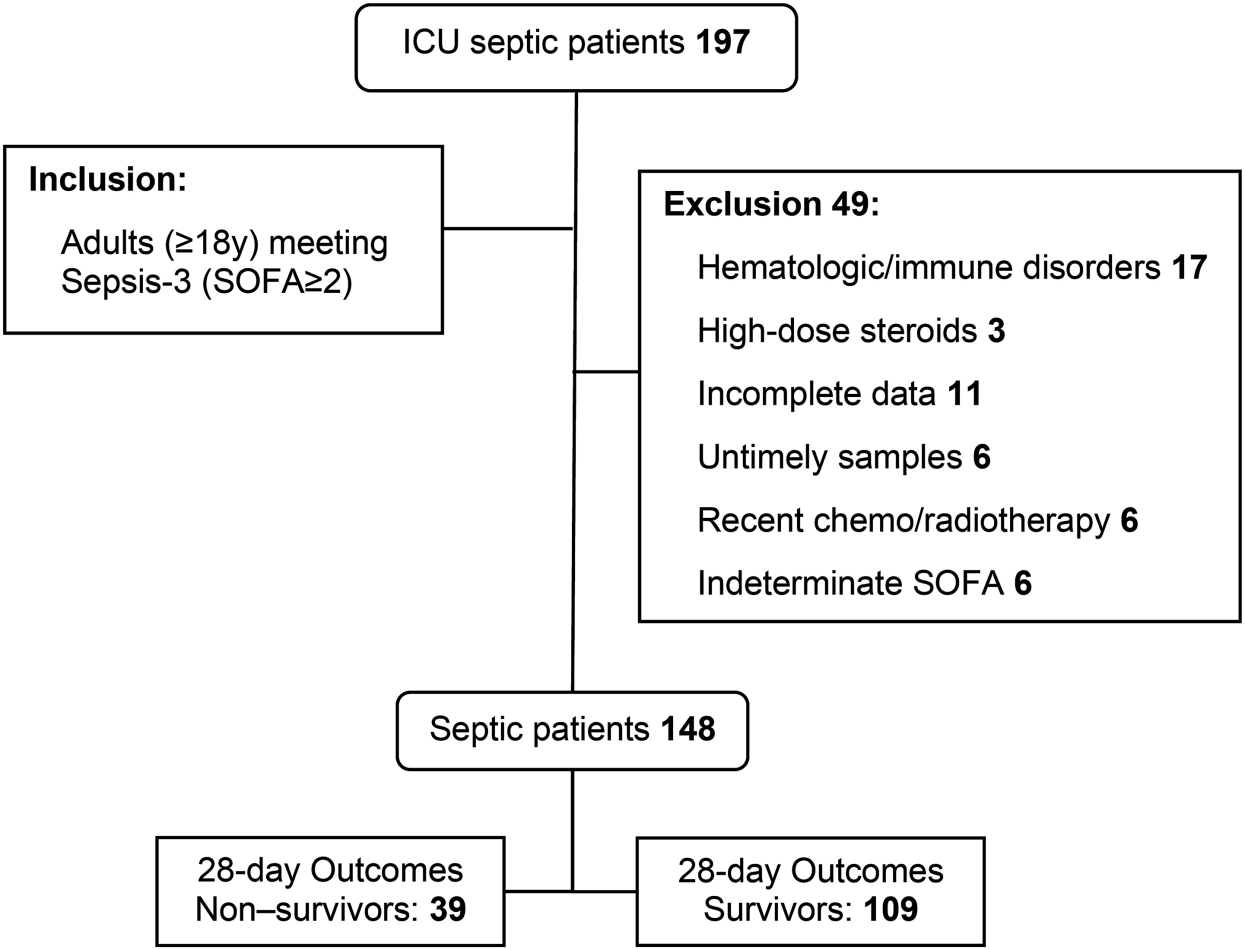
Flowchart of patient enrollment.

A total of 197 patients with sepsis were collected in this study, and 148 cases were finally included after the following exclusion criteria (Figure 1).

Excluded cases:

1. Patients with a comorbid history of hematologic disorders, including 5 cases of acute myeloid leukemia, 2 cases of aplastic anemia, and 1 case of diffuse large B-cell lymphoma; patients with a comorbid history of immunodeficiency, including 5 cases of systemic lupus erythematosus and 2 cases of dermatophytosis; and 2 cases of pulmonary infections associated with a history of HIV;
2. Enrollment of 3 patients with a recent history of receiving a large dose of hormone therapy (a large dose was defined as more than 1 mg/(kg·d) of prednisone or other equivalent dose of glucocorticoids over 7 days);
3. Of the patients with an unclear underlying SOFA score, there were 6 cases;
4. For multiple reasons, complete information was not available for 11 patients;
5. 6 cases in which relevant tests were not performed within 24 h of the patient’s admission to the hospital with a confirmed diagnosis of sepsis;
6. 6 cases who had a history of malignancy treated with radiotherapy or chemotherapy within 6 months before admission.

### 3.2 Clinical characteristics of patients

Patients with sepsis were categorized as surviving and dying based on their clinical outcome at 28 days. As can be seen from the following tables (Table 1, Supplementary Table S1), there was no significant difference between surviving and dying septic patients in terms of age and gender using the chi-square test and *t*-test. However, significant differences were presented in qSOFA score, SOFA score, APACHE II score, Lac, IL-10, lymphocyte, and ILR.

**Table 1:** Comparison of general characteristics between survivors and non-survivors in septic patients.

Items	Survival (*n* = 109)	Non-survival (*n* = 39)	*t* value/*Z* value	*p* value
Age (years)	62.98 ± 16.93	66.41 ± 14.86	−0.96	0.337
HR (beats/min)	103 ± 22	120 ± 22	−3.82	<0.001
RR (breaths/min)	22 (20, 26)	26 (23, 32)	−3.279	0.001
T (°C)	36.8 (36.5, 38.1)	36.7 (36.5, 38.1)	−0.682	0.495
qSOFA	1 (1, 2)	2 (1, 2)	−3.358	0.001
SOFA	4 (3, 6)	6 (4, 10)	−3.82	<0.001
APACHE II	23 (18, 28)	45 (25, 49)	−5.341	<0.001
GCS	15 (15, 15)	15 (13, 15)	−2.004	0.045
OI	300.68 ± 112.68	276.33 ± 144.69	1.467	0.142
Cr (umol/L)	101 (74, 172)	159 (108, 243)	−3.13	0.002
TBIL (umol/L)	17.20 (10.43, 30.08)	29.10 (13.50, 63.00)	−3.025	0.002
PLT (×10^9^/L)	145 (91, 225)	101.00 (32, 199)	−2.239	0.025
WBC (×10^9^/L)	11.54 (7.15, 16.97)	10.43 (4.64, 17.94)	−0.725	0.469
NEUT (×10^9^/L)	10.55 (6.34, 15.84)	9.28 (3.97, 17.03)	−0.783	0.434
PCT (ng/mL)	2.56 (0.87, 53.20)	13.94 (1.48, 48.8)	−1.396	0.163
PT (s)	15.3 (14.5, 16.9)	17.3 (15.8, 21.1)	−4.282	<0.001
APTT (s)	39.45 (34.68, 46.80)	44.7 (39.3, 55.5)	−2.848	0.004
PTA (%)	71.10 ± 16.31	56.25 ± 16.28	−4.367	<0.001
D-Dimer (ng/mL)	4.61 (2.25, 9.25)	5.06 (2.75, 17.94)	−0.958	0.338
Lac (mmol/L)	1.90 (1.00, 3.30)	3.25 (1.73, 8.18)	−3.563	<0.001
ALB (g/L)	29 (26, 32)	26 (23, 31)	−2.189	0.029
IL-6 (pg/mL)	132 (65, 312)	229 (104, 815)	−2.493	0.013
IL-10 (pg/mL)	9.96 (1.94, 33.08)	112.47 (29.80, 273.91)	−5.952	<0.001
Lym (×10^9^/L)	0.62 (0.41, 0.83)	0.28 (0.16, 0.39)	−5.916	<0.001
ILR	16.37 (3.11, 66.65)	302.33 (118.73, 972.52)	−6.583	<0.001

Continuous variables were analyzed using an independent samples *t*-test and Wilcoxon-Mann–Whitney U test to assess between-group differences. For normally distributed data, group comparisons were analyzed using independent *t*-tests, reported as mean ± SD (*x̄* ± s). For non-normally distributed data, the Wilcoxon-Mann–Whitney U test was used, reported as median (IQR) [M (IQR)]. HR, Heart rate; RR, Respiratory rate; T, Temperature; qSOFA, Quick Sequential Organ Failure Assessment; SOFA, Sequential Organ Failure Assessment; APACHE, II Acute Physiology and Chronic Health Evaluation II; GCS, Glasgow Coma Scale; OI, Oxygenation Index; Cr, Creatinine; TBIL, Total bilirubin; PLT, Platelet count; WBC, White blood cell count; NEUT, Neutrophil count; PCT, Procalcitonin; PT, Prothrombin time; APTT, Activated partial thromboplastin time; PTA, Prothrombin activity; Lac, Lactate; ALB, Albumin; IL-6, Interleukin-6; IL-10, Interleukin-10; Lym, Lymphocyte count; ILR, Interleukin-10 to lymphocyte ratio.

### 3.3 Feature importance ranking for septic mortality via SHAP value

**Figure 2:**
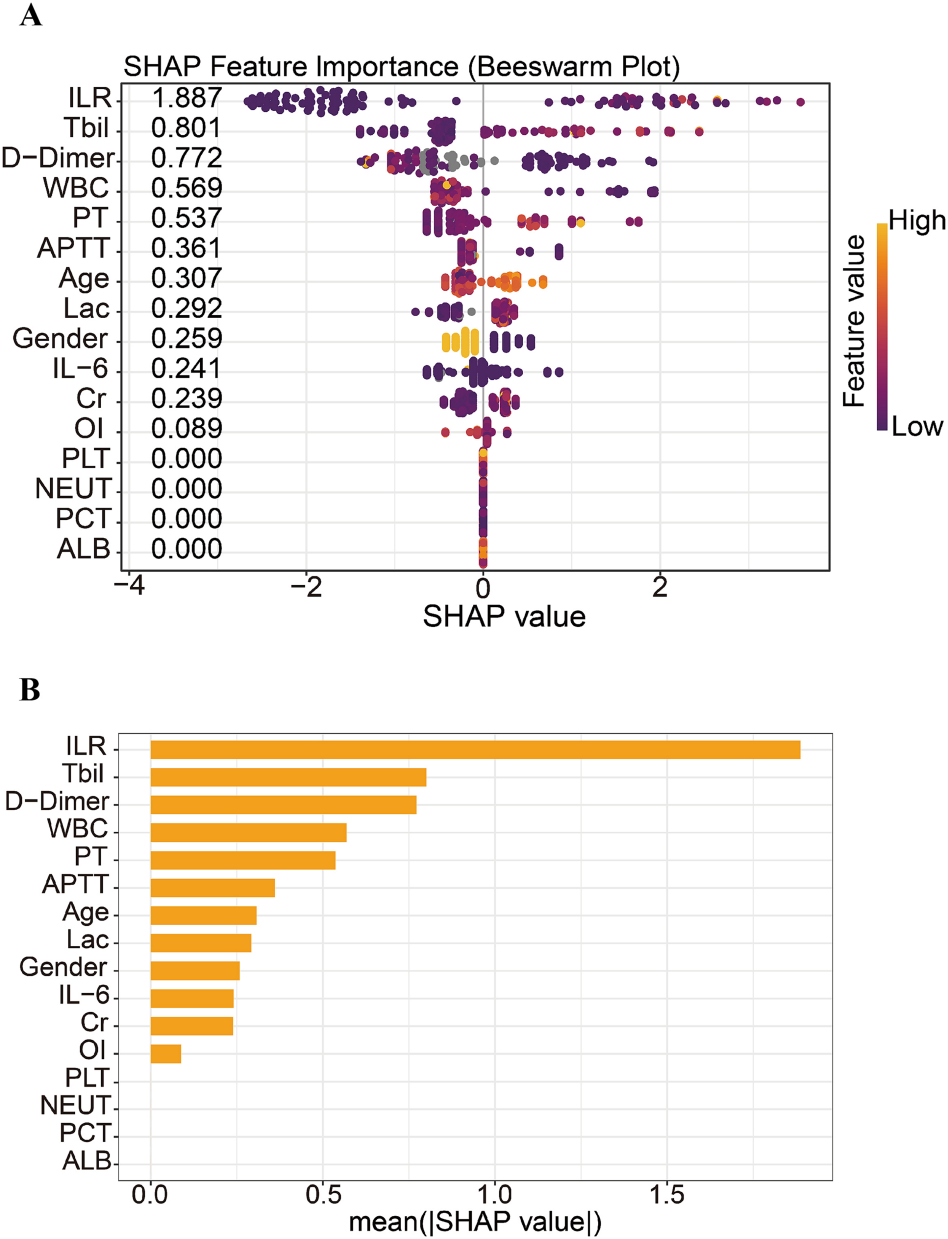
SHAP-based interpretation of model feature importance. **(A)** SHAP beeswarm plot illustrating the individual impact of each feature on the model output. Each dot represents a sample, with color indicating the feature value (purple = low, yellow = high). The ILR shows the strongest influence on predictions, followed by Tbil and D-dimer. **(B)** Bar plot of mean absolute SHA*p* values, summarizing the overall importance of each feature. ILR ranks highest, indicating its dominant role in the model’s predictive power.

We used R language to construct XGBoost machine learning model based on objective laboratory indicators, literature support parameters and commonly used clinical indicators, and applied SHAP (Shapley Additive Explanations) value analysis to analyze the key predictors of prognosis of death in septic patients (Figure 2) and the results of their model performance evaluation (Supplementary Table S2). The SHAP analysis showed that the ILR had the highest contribution to the model output (mean SHAP value = 1.887), indicating that this metric has a significant impact on mortality risk prediction. This was followed by total bilirubin (Tbil, SHAP value = 0.801) and D-dimer (SHAP value = 0.772). Other statistically significant predictors included white blood cell count (WBC), prothrombin time (PT), activated partial thromboplastin time (APTT), patient age, and Lac levels.

### 3.4 Multivariate logistic regression analysis

Based on the results of SHAP analysis, the present study adopted a holistic assessment strategy in the selection of variables: to avoid the local bias that may be caused by individual organ function indexes (e.g., coagulation parameters), a comprehensive organ function assessment system such as SOFA scores and APACHE II scores was turned to as a substitute. This design aims to more comprehensively reflect the overall characteristics of multi-organ dysfunction in sepsis. We used age, ILR, SOFA, APACHE II, Lac, IL-6, D-dime, albumin (ALB), and underlying diseases (e.g., diabetes mellitus and hypertension) as independent variables, and survival status of septic patients (survival = 1, death = 0) as the dependent variable, and performed multivariate logistic regression analysis. Stepwise regression was used for this analysis (Table 2). The results showed that APACHE II score (OR = 1.143, 95% confidence interval CI 1.060–1.232) and ILR (OR = 1.005, 95% CI 1.001–1.009) were identified as independent risk factors for death, whereas Lac also demonstrated a dominance ratio OR = 1.047. specifically. The risk of death in patients with sepsis increased significantly with increasing APACHE II score and ILR values.

**Table 2:** Multivariate logistic regression analysis of risk factors for 28-day mortality in septic patients.

	OR	*p* value	95%CI	
Age	1.017	0.510	0.968	1.067
HTN	0.69	0.685	0.115	4.133
DM	0.382	0.328	0.056	2.629
SOFA	1.028	0.860	0.757	1.395
APACHE II	1.143	<0.001	1.060	1.232
ILR	1.005	0.013	1.001	1.009
Lac	1.047	0.786	0.751	1.460
IL-6	1	0.452	1.000	1.000
D-Dimer	0.988	0.588	0.947	1.031
ALB	0.985	0.876	0.818	1.187

HTN, Hypertension; DM, Diabetes mellitus; OR, Odds ratio; CI, Confidence interval; other abbreviations are as same as Table 1.

### 3.5 Cox proportional hazards regression analysis

Cox proportional hazards regression analyses were performed with age, ILR, SOFA, APACHE II, Lac, IL-6, D-dimer, ALB, and underlying disease (diabetes mellitus, hypertension) as the independent variables, and time to death within 28 days of admission in septic patients as the dependent variable, respectively (Table 3). Cox regression showed that APACHE II score (HR = 1.102, 95% CI 1.046–1.161) and ILR (HR = 1.239, 95% CI 1.097–1.399) were independent predictors of death. For every 1-point increase in APACHE II score, the risk of death increased by 10.2%. For every 1-unit increase in ILR, the risk of death increased by 23.9%.

**Table 3:** Cox proportional hazards regression analysis of predictors for 28-day survival in septic patients.

	HR	*p* value	95%CI	
Age	1.003	0.882	0.967	1.040
HTN	0.547	0.235	0.202	1.481
DM	0.772	0.607	0.289	2.063
SOFA	0.971	0.778	0.790	1.193
APACHE II	1.102	0.000	1.046	1.161
ILR	1.239	0.001	1.097	1.399
Lac	1.068	0.428	0.908	1.257
IL-6	1.000	0.578	1.000	1.000
D-Dimer	1.000	0.982	0.976	1.024
ALB	1.020	0.711	0.919	1.131

HR, hazard ratio; CI, Confidence interval; other abbreviations are as same as Table 1.

### 3.6 ROC for predicting prognosis in septic patients

**Figure 3:**
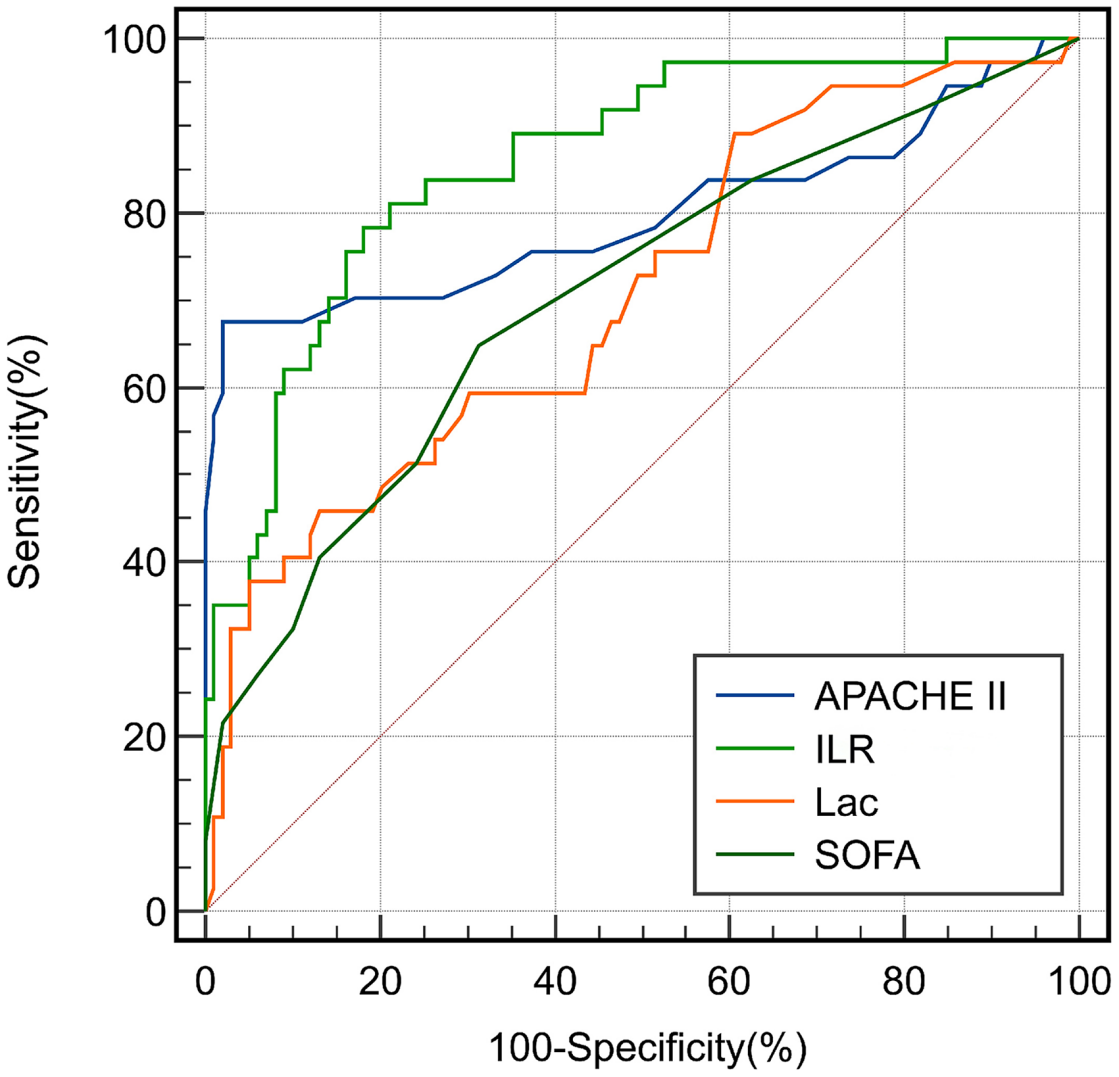
ROC for 28-day mortality in septic patients.

Based on the results of multivariate logistic regression analysis and Cox proportional hazards regression, we further evaluated the predictive efficacy of each independent predictor for the 28-day risk of death in patients with sepsis. Based on the significance levels of OR (Logistic regression) and HR (Cox regression), we selected four key indicators - ILR, Lac, SOFA score, and APACHE II score, for receiver operator characteristic curves (ROCs) analysis. The predictive efficacy of ILR, Lac, SOFA score, and Apache II score for death in patients with sepsis is shown in Figure 3 and Table 4. The area under the ROC curve (AUC) for ILR is 0.860 (95% CI 0.791–0.914, *p* < 0.001), the highest efficacy, with a sensitivity of 79.49% and a specificity of 81.65% at an optimal cutoff value of 97.4 (Supplementary Figure S1). The area under the ROC curve (AUC) for Lac was 0.706 (95% CI 0.621–0.781, *p* < 0.001), with a cutoff value of 4.1 score corresponding to a 46.15% sensitivity and 85.98% specificity (Supplementary Figure S2). The area under the ROC curve (AUC) for SOFA was 0.704 (95% CI 0.624–0.777, *p* < 0.001), and its cutoff value of 4 points corresponded to a sensitivity of 71.79% and a specificity of 59.63% (Supplementary Figure S3). The area under the ROC curve (AUC) for Apache II was 0.797 (95% CI 0.720–0.861, *p* < 0.001), and its cutoff value of 38 points corresponded to a sensitivity of 67.57% and a specificity of 98.02% (Supplementary Figure S4).

**Table 4:** ROC analysis for mortality prediction in sepsis.

Index	AUC	*P* value	95% CI	Sensitivity (%)	Specificity (%)	Cutoff value
ILR	0.860	<0.001	0.791–0.914	79.49	81.65	97.4
SOFA	0.704	<0.001	0.624–0.777	71.79	59.63	4
Lac	0.706	<0.001	0.621–0.781	46.15	85.98	4.1
APACHE II	0.797	<0.001	0.720–0.861	67.57	98.02	38

AUC area under the receiver operating characteristic curve.

### 3.7 ILR-lac risk stratification and multimethod prognostic validation in sepsis

**Figure 4:**
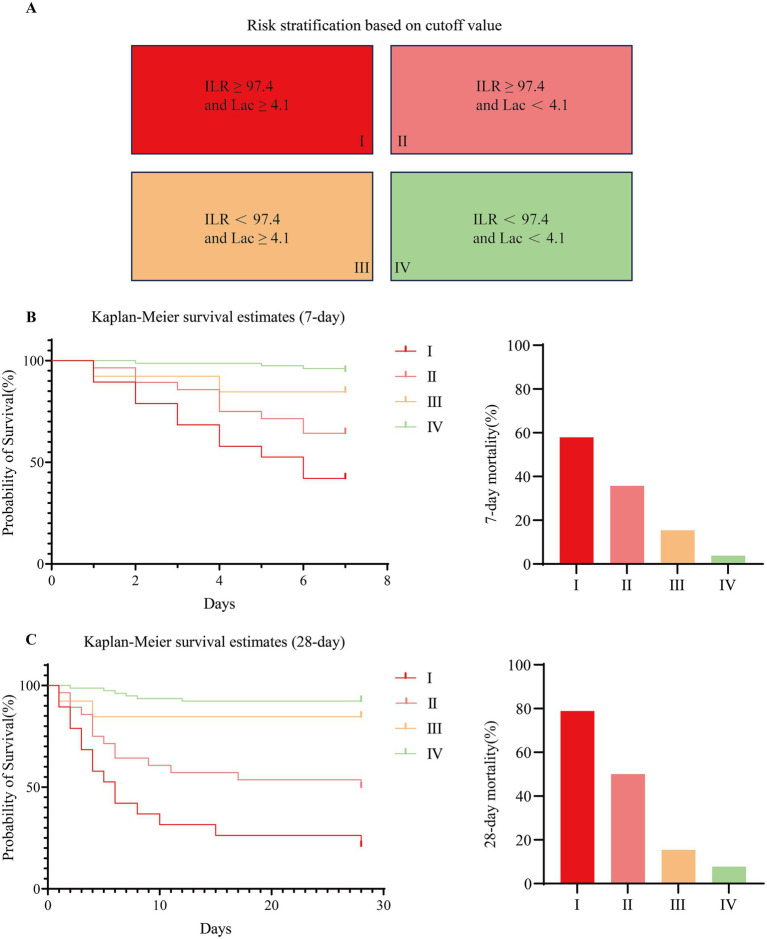
Risk stratification and survival analysis by ILR and Lactate (Lac) optimal cutoff value in sepsis. **(A)** Risk stratification criteria: Level I: ILR ≥ 97.4 and Lac ≥4.1 mmol/L; Level II: ILR ≥ 97.4 and Lac <4.1 mmol/L; Level III: ILR < 97.4 and Lac ≥4.1 mmol/L; Level IV: ILR < 97.4 and Lac <4.1 mmol/L. **(B)** 7-day and **(C)** 28-day Kaplan–Meier survival curves showing significant mortality differences across levels (I > II > III > IV, all log-rank *p* < 0.001).

Using ROC-derived optimal cutoffs (ILR: 97.4; Lac: 4.1 mmol/L), patients were stratified into four risk stratification levels (Levels I–IV), where Level I represented the highest-risk stratum and Level IV the lowest. Kaplan–Meier survival analyses at 7 and 28 days revealed significant intergroup differences (7-day: χ^2^ = 37.84; 28-day: χ^2^ = 61.41; both *p* < 0.001) (Supplementary Tables S3, S4), with mortality rates demonstrating a consistent hierarchy across timepoints: 57.89, 35.71, 15.38, and 3.85% at 7 days, progressing to 78.95, 50.00, 15.38, and 7.69% at 28 days in the respective risk stratification levels (Figure 4). In addition, trend analysis of survival analysis Log-Rank test (7-day *χ*^2^ = 36.21, *p* < 0.001; 28-day *χ*^2^ = 56.62, *p* < 0.001) further confirmed the statistically significant trend of deterioration in patient survival with increasing level rank. This trend analysis is particularly useful for assessing consistent patterns of change in survival with level rank. To further validate differences between survival curves, we used the Gehan-Breslow-Wilcoxon test, which is more sensitive to early events. The results (7-day *χ*^2^ = 37.79, *p* < 0.001; 28-day *χ*^2^ = 58.82, *p* < 0.001) support the conclusion that there is a significant difference between the survival curves and suggest that this difference may have been apparent at an early stage of follow-up. This dual stratification method, based on objective cutoff values, ensures standardization of assessment and more precise identification of different risk stratification levels, providing a reliable basis for early clinical intervention.

## 4 Discussion

The pathophysiological mechanism of sepsis, as a life-threatening organ dysfunction caused by a dysregulated systemic inflammatory response triggered by infection, involves a complex dysregulation of the immune response characterized by a dynamic imbalance between an early excessive pro-inflammatory response and a subsequent state of immunosuppression. This imbalance not only exacerbates organ dysfunction but is also strongly associated with high patient mortality (22, 23). The immunosuppressive state of sepsis is characterized by an overproduction of anti-inflammatory cytokines (e.g., IL-10) and a significant decrease in the number of lymphocytes, which together constitute the core features of immune dysfunction in septic patients. This immunosuppressive state leads to decreased clearance of pathogens and increased risk of secondary infections in patients, making it an important driver of sepsis mortality (24). Studies have shown that elevated levels of IL-10, an important immunomodulatory factor that inhibits the release of pro-inflammatory cytokines and impairs effector T-cell function, are strongly associated with poor prognosis in patients with sepsis (25, 26). On the other hand, lymphopenia reflects extensive depletion of immune cells and is another key manifestation of immunosuppression in sepsis. In addition, elevated levels of Lac, a marker of tissue hypoperfusion and metabolic disturbances, not only suggest organ dysfunction but may also exacerbate immunosuppressive symptoms by affecting immune cell function (27).

The central feature of immunosuppression in sepsis is well characterized by the dynamic changes in the ILR. The overexpression of IL-10, a key mediator of immunomodulation, not only attenuates T-cell activation by inhibiting the expression of MHC-II class II molecules and co-stimulatory signals (e.g., CD80/CD86) in antigen-presenting cells (APCs), but also directly induces regulatory T-cells (Tregs) expansion, further reinforcing the immunosuppressive microenvironment (28). At the same time, lymphopenia reflects the widespread phenomenon of apoptosis of immune cells in sepsis, especially the significant depletion of CD4 + T cells and B cells (29). Lac, as a reflection of tissue hypoperfusion and metabolic disturbances, not only suggests circulatory disturbances but also further impairs immune effector cell activity by impairing mitochondrial function. It has been shown that high Lac levels promote Treg expansion (30) and inhibit CD8 + T-cell function (31), creating a cycle of immunosuppression that is mutually reinforcing with IL-10. Therefore, the combination of ILR and Lac may reflect the pathological state from both immunologic and metabolic aspects.

In this study, the ILR (302.33 [118.73, 972.52]) and Lac (3.25 [1.73, 8.18]) were significantly higher in the patients in the death group than in the survivor group (16.37 [3.11, 66.65]), and (1.90 [1.00, 3.30]), and the difference was statistically significant (*p* < 0.001). Both multivariate logistic regression analysis (OR = 1.005, 95% CI 1.001–1.009) and Cox proportional hazards regression (HR = 1.239, 95% CI 1.097–1.399) indicated that the ILR was an independent risk factor for 28-day mortality. This ratio not only reflects the intensity of the anti-inflammatory response (IL-10), but also quantifies the depletion of immune cells (lymphocyte), which is highly compatible with the “high-inflammatory-high-immunosuppressive” feature of sepsis (32). Compared with traditional scoring systems such as SOFA and APACHE II, ILR showed superior predictive efficacy in this study. Its ROC curve area under the curve was 0.860, which was better than that of APACHE II (0.797) and SOFA score (0.704), and it had good sensitivity (79.49%) and specificity (81.65%) at the cutoff value of 97.4. Importantly, ILR not only outperformed SOFA and APACHE II in predictive accuracy but also provided incremental prognostic value by capturing the immunosuppressive component of sepsis, which is insufficiently addressed by conventional scoring systems. This suggests that ILR may serve as a useful complement to existing tools, enhancing risk stratification and guiding individualized management strategies in septic patients.

A previous study investigated the ILR in patients with severe sepsis and identified an optimal cutoff value of 23.39 ng/ml^2^ for predicting 28-day mortality (33). In contrast, the cutoff value identified in our study was 97.4 pg/10^9^. This discrepancy may be attributed to several factors: (1) Differences in the enrolled patient population and sample size. The study by Li et al. focused specifically on patients with severe sepsis, including 63 patients, among whom 20 were non-survivors. Our study included a broader spectrum of septic patients (including those with septic shock) with a larger sample size of 148 patients and 39 non-survivors. The inclusion of a more generalized sepsis population and a larger sample size in our study may enhance the generalizability of our findings. (2) Differences in ILR measurement, including both the units used and laboratory assay techniques. Li et al. reported ILR in ng/ml^2^, whereas our study utilized pg/10^9^. Variations in IL-10 assay methods across laboratories may further contribute to differences in reported values. Both the differences in enrolled populations and in measurement methods/units are important factors that may underlie the observed discrepancy in cutoff values. These observations highlight the need for future studies with larger, multicenter cohorts to optimize and validate universally applicable cutoff values.

Compared with previous studies, the integrated design of the ILR compensates for the limitations of a single indicator. For example, although elevated IL-10 levels are associated with immunosuppression, their individual differences and detection time may affect the generalizability of the thresholds (34); whereas lymphopenia, although reflective of immune depletion, does not allow for a comprehensive assessment of the dynamic balance between inflammation and immunosuppression (35). By simultaneously quantifying both the levels of anti-inflammatory factors and the degree of immune cell depletion, the ILR more accurately captures the severity of immune imbalance in sepsis, thus providing a more reliable basis for clinical decision-making. This finding was also validated by the SHAP machine learning model, which ranked first among all features, indicating its critical role in prognostic prediction.

Of interest, the present study found that the median IL-10 in the patients in the death group (112.47 pg/mL) was more than 11-fold higher than that in the survival group (9.96 pg/mL), while the lymphocyte counts were decreased by 55% (0.28 vs. 0.62 × 10^9^/L), presenting a significant immune imbalance or suggesting that the body has moved from an immune compensatory The ILR acutely captured this critical turn by integrating anti-inflammatory intensity with immune cell reserve, and predicted efficacy (AUC = 0.860) better than the SOFA score (AUC = 0.704). This result is consistent with recent sepsis immunophenotyping studies, suggesting that a high ILR may represent an “immunosuppressive” subtype that may benefit from targeted immunomodulatory therapy (36, 37).

In recent years, multiple sepsis typing strategies have been proposed to cope with its high degree of heterogeneity. For example, clinical data-based SENECA typing (types α, β, γ, and δ) identifies patient subgroups through electronic health record variables, but its distribution and mortality rates vary significantly across cohorts (e.g., δ types accounted for 26–48% of the MARS and NICE cohorts but only 13% of the MIMIC-IV cohort) (38), suggesting that its generalizability may be limited by population and geographic location. In addition, typing based on vital sign trajectories (e.g., group A with hyperthermia with hypotension versus group D with advanced age and hypothermia), although predictive of therapeutic response (e.g., group D is more sensitive to balanced crystalloid fluids) (39), requires continuous ambulatory monitoring and high complexity of clinical implementation. Transcriptomic approaches (e.g., Mars1-4 and SRS1-2 typing) define immunosuppressive or hyperinflammatory states (e.g., SRS1 is associated with T-cell depletion and a significantly higher mortality rate) through gene expression profiles (40, 41), but their reliance on whole-genome sequencing and bioinformatic analyses is costly and difficult to apply in real time, even when using PCR to detect the specific RNA of the target type. Despite the value of these approaches in mechanism exploration and precision therapy, their complexity (e.g., AI models require multi-parameter integration) and low tractability (e.g., lack of immediate detection tools) limit clinical dissemination (42–44).

In contrast, the ILR and Lac stratification strategy proposed in this study offers significant advantages: (1) Operational Simplicity: Risk stratification requires only three routine tests (IL-10, lymphocyte count, and Lac), enabling real-time bedside assessment without complex calculations or continuous monitoring. This facilitates immediate clinical decision-making, particularly in resource-limited settings; (2) Cost-Effectiveness: By minimizing blood sample volume (≤2 mL) and utilizing low-cost tests (versus transcriptomics or AI models), this approach reduces financial and logistical burdens for patients and healthcare systems; (3) Pathophysiology Integration: The dual assessment of immunosuppression (ILR) and tissue hypoperfusion (Lac) provides superior risk stratification and prognosis prediction compared to single-dimension tools, with significant mortality gradient; (4) Immediate Decision Support: Defined cutoff values (ILR ≥ 97.4, Lac ≥4.1 mmol/L) allow direct clinical application, eliminating reliance on external algorithms. This supports rapid intervention adjustments, especially in economically disadvantaged regions. Future studies could explore the joint application of ILR with existing typing (e.g., SENECA or SRS) to further optimize precision management of sepsis.

However, this study still has some limitations. First, the single-center retrospective design may have led to selection bias, and the small sample size (*n* = 148) may have limited statistical power. Second, the non-inclusion of other potential modifiers [e.g., PD-1/PD-L1 expression (45) or metabolic markers (46, 47)] or external validation cohorts may affect the generalizability of the results. Future multicenter, prospective studies are needed to further validate the predictive efficacy of the ILR and Lac and to explore their association with immunomodulatory therapy. For example, whether intervention strategies targeting the IL-10 pathway or lymphocyte reconstitution can improve the prognosis of high-risk patients deserves in-depth investigation. In addition, the standardization of ILR and Lac assays and the clinical applicability of cutoff values still need to be further optimized. Third, this study only assessed a limited number of prognostic markers (ILR and lactate), whereas many other factors such as age, infection site, and baseline organ function are also known to influence outcomes in sepsis. Although incorporating a broader set of variables or using AI-based predictive models could potentially improve predictive accuracy, such approaches may increase costs and reduce feasibility for bedside application. Our current strategy emphasizes simplicity and clinical operability, but inevitably sacrifices some precision compared with large-scale data-driven models.

## 5 Conclusion

ILR and Lac synergistically combine immunologic profiling with tissue perfusion assessment, establishing a biomarker-based stratification system that demonstrates significant prognostic discrimination. Our proposed four-tier risk stratification (using cutoffs of ILR ≥ 97.4 and Lac ≥ 4.1 mmol/L) showed markedly distinct mortality outcomes (28-day: 78.95–7.69%), its operational simplicity is superior to complex AI models and circumvents the difficulty of clinical implementation of SOFA/APACHE II, etc. Future multicenter studies are needed to validate the generalizability of the cutoff values and to explore targeted therapeutic strategies for high-risk stratification to promote its widespread use in clinical practice.

## Data Availability

The raw data supporting the conclusions of this article will be made available by the authors, without undue reservation.
