# Supplementary material for: IL-10 to lymphocyte ratio (ILR) and lactate in the prognosis prediction and risk stratification of sepsis: a pilot study
Source: Front Med (Lausanne). 2025 Sep 10;12:1665915. doi: 10.3389/fmed.2025.1665915 (PMC12457667; doi:10.3389/fmed.2025.1665915)
Supplement: Supplementary file 1 [file Data_Sheet_1.docx]

**Supplementary Materials of “IL-10 to Lymphocyte Ratio (ILR) and Lactate in the Prognosis Prediction and Risk Stratification of Sepsis: A Pilot Study”**





**Supplementary Figure 1: ROC curve for ILR in predicting 28-day mortality in septic patients.** The blue-shaded area represents the 95% confidence interval (CI) of the ROC curve. The area under the curve (AUC) was 0.860 (*p* < 0.001). The optimal cutoff value for ILR was 97.4 (sensitivity: 79.49%; specificity: 81.65%).





**Supplementary Figure 2:** **ROC curve for lactate (Lac) in predicting 28-day mortality in septic patients.** The blue-shaded area represents the 95% confidence interval (CI) of the ROC curve. The area under the curve (AUC) was 0.706 (*p* < 0.001). The optimal cutoff value for Lac was 4.1 mmol/L (sensitivity: 46.15%; specificity: 85.98%).





**Supplementary Figure 3: ROC curve for SOFA score in predicting 28-day mortality in septic patients.** The blue-shaded area represents the 95% confidence interval (CI) of the ROC curve. The area under the curve (AUC) was 0.704 (*p* < 0.001). The optimal cutoff value for SOFA was 4 (sensitivity: 71.79%; specificity: 59.63%).





**Supplementary Figure 4: ROC curve for AAPACHE II score in predicting 28-day mortality in septic patients.** The blue-shaded area represents the 95% confidence interval (CI) of the ROC curve. The area under the curve (AUC) was 0.797 (*p* < 0.001). The optimal cutoff value for AAPACHE II was 38 (sensitivity: 67.57%; specificity: 98.02%).

|  | Group | Survival (n=109) | Non-survival (n=39) | *χ2* value | *p* value |
| --- | --- | --- | --- | --- | --- |
| Sex, n (%) | Female | 41(37.615) | 19(48.718) | 1.469 | 0.226 |
|  | Male | 68(62.385) | 20(51.282) | |  |
| Hypertension, n (%) | - | 8(7.339) | 2(5.128) | 0.235 | 0.889 |
|  | No | 59(54.128) | 22(56.410) | |  |
|  | Yes | 42(38.532) | 15(38.462) | |  |
| Coronary artery disease, n (%) | - | 8(7.339) | 2(5.128) | 1.446 | 0.485 |
|  | No | 93(85.321) | 36(92.308) | |  |
|  | Yes | 8(7.339) | 1(2.564) |  |  |
| Chronic kidney disease, n (%) | - | 8(7.339) | 2(5.128) | 1.359 | 0.507 |
|  | No | 98(89.908) | 37(94.872) | |  |
|  | Yes | 3(2.752) | 0(0.000) |  |  |
| Diabetes mellitus, n (%) | - | 8(7.339) | 2(5.128) | 0.672 | 0.715 |
|  | No | 62(56.881) | 25(64.103) | |  |
|  | Yes | 39(35.780) | 12(30.769) | |  |
| Chronic obstructive pulmonary disease, n (%) | - | 8(7.339) | 2(5.128) | 1.749 | 0.417 |
|  | No | 97(88.991) | 37(94.872) | |  |
|  | Yes | 4(3.670) | 0(0.000) |  |  |
| Chronic liver disease, n (%) | - | 8(7.339) | 2(5.128) | 1.364 | 0.506 |
|  | No | 99(90.826) | 35(89.744) | |  |
|  | Yes | 2(1.835) | 2(5.128) |  |  |

**Supplementary Table 1. Comparison of general characteristics between survivors and non-survivors in septic patients.** Note: Categorical variables were compared between groups using the chi-square test, with results presented as frequencies and percentages. Ten patients had unclear or missing data regarding their medical history ("-" category) and were excluded from the respective subgroup analyses for each comorbidity. This missing data was uniformly distributed across both survival (n=8) and non-survival (n=2) groups.

| Dataset | AUC | 95%CI |  |
| --- | --- | --- | --- |
| Train | 1 | 1 | 1 |
| Test | 0.724 | 0.555 | 0.893 |

**Supplementary Table 2. Model performance based on SHAP analysis**. Note: To evaluate the predictive performance of the XGBoost model in distinguishing survival outcomes among sepsis patients, we calculated the area under the receiver operator characteristic curve (AUC) along with the corresponding 95% confidence intervals (CI) for both the training and testing datasets. In the training set, the model achieved an AUC of 1.000 (95% CI: 1.000–1.000), indicating perfect classification of the training samples. However, in the independent test set, the AUC dropped to 0.724 (95% CI: 0.555–0.893), reflecting only moderate predictive performance. The substantial discrepancy between the training and test AUC values suggests that the model may have overfitted the training data — that is, it captured patterns specific to the training set that do not generalize well to unseen data. This overfitting could be attributed to a limited sample size or imbalanced variable distributions. Further optimization, including hyperparameter tuning, regularization strategies, or the use of ensemble models, may help improve model robustness and generalizability in future studies.

|  | *χ2* value | df | *p* value |
| --- | --- | --- | --- |
| Log-rank (Mantel-Cox) test | 37.84 | 3 | <0.001 |
| Log-rank test for trend | 36.21 | 1 | <0.001 |
| Gehan-Breslow-Wilcoxon test | 37.79 | 3 | <0.001 |

**Supplementary Table 3. 7Day-Survival Curve Analysis-Significance Testing Results.** Note: *df* Degrees of freedom*.*

|  | *χ2* value | df | *p* value |
| --- | --- | --- | --- |
| Log-rank (Mantel-Cox) test | 61.41 | 3 | <0.001 |
| Log-rank test for trend | 56.62 | 1 | <0.001 |
| Gehan-Breslow-Wilcoxon test | 58.82 | 3 | <0.001 |

**Supplementary Table 4. 28Day-Survival Curve Analysis-Significance Testing Results.** Note: *df* Degrees of freedom*.*
